# Clinicopathologic factors linked to oncologic outcomes for renal cell carcinoma with sarcomatoid dedifferentiation: A PRISMA-compliant systematic review and meta-analysis

**DOI:** 10.3389/fsurg.2022.922150

**Published:** 2022-10-21

**Authors:** Lisong Shan, Xue Shao, Liangyou Gu, Minhong Wu, Pengxiu Lin, Zhiling Yu, Qingsheng Chen, Daqing Zhu

**Affiliations:** ^1^ Department of Urology, Hainan Hospital, Chinese PLA General Hospital, Sanya, China; ^2^ Department of Neurology, Hainan Hospital, Chinese PLA General Hospital, Sanya, China; ^3^ Department of Urology, The Third Medical Centre, Chinese PLA General Hospital, Beijing, China; ^4^ Department of Urology, Yichun People's Hospital, Yichun, China

**Keywords:** renal cell carcinoma, sarcomatoid differentiation, prognosis, meta-analysis, surgical oncology

## Abstract

**Background:**

There are still differences in the prognostic factors of renal cell carcinoma with sarcomatoid dedifferentiation (sRCC). The aim of this study was to evaluate important predictors of survival in patients with sRCC.

**Patients and methods:**

A comprehensive search of PubMed, Embase, and Cochrane Library was conducted to identify eligible studies. The endpoints embraced overall survival (OS), cancer-specific survival (CSS), and progression-free survival (PFS). Hazard ratios (HRs) and related 95% confidence intervals (CIs) were extracted.

**Results:**

A total of 13 studies were included for analyses. The pooled results showed that high European Cooperative Oncology Group performance score (HR 2.39, 95% CI 1.32–4.30; *P* = 0.004), high T stage (HR 2.18, 95% CI 1.66–2.86; *P* < 0.001), positive lymph node (HR 1.54, 95% CI 1.40–1.69; *P* < 0.001), distant metastasis (HR 2.52, 95% CI 1.99–3.21; *P* < 0.001), lung metastases (HR 1.45, 95% CI 1.16–1.80; *P* < 0.001), liver metastases (HR 1.71, 95% CI 1.30–2.25; *P* < 0.001), tumor necrosis (HR 1.78, 95% CI 1.14–2.80; *P* = 0.010), and percentage sarcomatoid ≥50% (HR 2.35, 95% CI 1.57–3.52; *P* < 0.001) were associated with unfavorable OS. Positive lymph node (HR 1.57, 95% CI 1.33–1.85; *P* < 0.001) and high neutrophil to lymphocyte ratio (HR 1.16, 95% CI 1.04–1.29; *P* = 0.008) were associated with unfavorable CSS. High T stage (HR 1.93 95% CI 1.44–2.58; *P* < 0.001) was associated with unfavorable progression-free survival.

**Conclusions:**

A meta-analysis of available data identified important prognostic factors for CSS, OS, and PFS of sRCC, which should be systematically evaluated for patient counseling, risk stratification, and treatment selection.

**Systematic Review Registration:**

https://www.crd.york.ac.uk/PROSPERO/display_record.php?RecordID=249449.

## Introduction

Renal cell carcinoma (RCC) represents about 3% of all adult malignances, and its global incidence has increased by about 2% a year over the past 20 years (1). Sarcomatoid dedifferentiation characterized by malignant spindle cells is rare in RCC and occurred in about 1%–8% of RCC patients (2). Since sarcomatoid dedifferentiation can occur in all kinds of RCC subtypes in histology, RCC with sarcomatoid dedifferentiation (sRCC) is no more believed to be a specific subtype (3, 4). Patients with sarcomatoid dedifferentiation tend to have a poor prognosis compared to other high-stage RCC patients. They represent 20% of cases with advanced disease (5, 6). In addition, sarcomatoid dedifferentiation has been reported to be correlated to an around 60% increased risk of cancer-specific mortality in cases with Fuhrman grade 4 disease (4).

The study of prognostic predictors of sRCC is of great significance in guiding postoperative patient consultation, risk stratification, and treatment selection. In recent decades, TNM staging system has remained the most widely applied staging system for RCC. Nevertheless, significant survival heterogeneity was identified in sRCC patients with the same TNM stage. Therefore, another prognostic model is needed to better individualize outcomes in patients with sRCC. By far, a number of studies have examined various clinical and pathological variables as prognostic factors in patients with sRCC. Several significant prognostic factors have been found, including T stage, lymph node status, distant metastasis, histology type, percentage of sarcomatoid dedifferentiation, and blood markers (2–4, 7–15). Moreover, several prognostic models have been developed to accurately predict tumor prognosis in each patient with sRCC (4, 11, 14).

However, most studies that have attempted to identify prognostic predictors of sRCC have been limited to small sample sizes, single-center designs, and nonhomogeneous populations (4, 14). The general applicability of the proposed prediction model cannot be guaranteed. Moreover, prognostic roles for patients with sRCC reported in the literature remain controversial. For these reasons, we aimed to evaluate important predictors of survival in RCC tumors with sarcomatoid dedifferentiation through a systematic review of relevant studies and a meta-analysis of available data.

## Patients and methods

The work has been reported in line with PRISMA (reporting checklist in Supplementary data) Guidelines. The protocol was registered in PROSPERO (ID: CRD42021249449).

### Search strategy

We conducted a computerized literature search of PubMed, Embase, and Cochrane Library through October 2021 to identify studies that focus on tumor prognosis in sRCC. The prognostic roles of sRCC have been analyzed in these studies.

Separate search of each database was performed with the following search items through MeSH headings, keywords, and text words: (“kidney cancer” or “renal tumor” or “renal cell carcinoma” or “renal cancer”) and (“sarcomatoid dedifferentiation” or “sarcomatoid differentiation” or “sarcomatoid” or “sarcomatoid component”) and (“survival” or “prognosis” or “outcome” or “recurrence” or “progression” or “mortality”). In addition, references to relevant studies were checked. These studies included original research, reviews, and letters or comments.

### Inclusion criteria and study eligibility

This study included patients pathologically diagnosed with renal cell carcinoma with sarcomatoid dedifferentiation. Literature studies focusing on prognostic factors for sRCC were included. The endpoints of oncologic outcomes embraced overall survival (OS), cancer-specific survival (CSS), and progression-free survival (PFS). Exclusion criteria were as follows: (1) cell or animal research; (2) studies that did not focused on RCC; (3) nonoriginal research (reviews, meta-analyses, letters to editor, comments); (4) case report or case series with less than 20 patients; (5) studies that included patients without sarcomatoid dedifferentiation; and (6) studies that did not report adjusted hazard ratios (HRs) and related 95% confidence intervals (CIs) based on multivariate analyses. When two or more studies examined the same variable at the same endpoint, the results were combined. Two authors independently reviewed the title and abstract to evaluate the full-text studies. Differences were resolved by discussion with senior authors.

The study quality was assessed using the Newcastle-Ottawa Scale, as recommended by the Cochrane Collaboration (16). Using a scoring system, each literature study was assessed according to three aspects, namely, criteria, comparability between groups, and identification of results of interest. Confirmation of evidence was scored using the Grading of Recommendations Assessment, Development and Assessment (GRADE) scoring system (17), which includes five criteria: study design, risk of bias, inconsistency and accuracy of results, and indirectness. The certainty of the evidence in each meta-analysis comes down to four levels.

### Data extraction

The authors extracted data from each included literature study separately. Any disagreement was resolved in consultation with senior authors. First, the overall characteristics of sRCC were evaluated by collecting baseline data, including first author name, study period and design, country, number of included patients, patient age, tumor stage, endpoints, and median follow-up period. Then, the HRs and 95% CIs of prognostic factors were extracted from multivariate analyses and cumulative analyses were performed.

### Statistical analysis

The predictive effect and its corresponding standard error were used to perform a meta-analysis of each possible factor for tumor prognosis. The cumulative effect of interest factors was evaluated by the inverse variance method. Cochrane *Q* and *I*^2^ statistics were used to evaluate statistical heterogeneity. If Cochran *Q* test obtained *P* values less than 0.05 or *I*^2^ statistics greater than 50%, significant heterogeneity was identified among studies. Otherwise, low-intermediate heterogeneity was identified. Taking into account insufficient studies and sample size, random effect has been applied for all the analyses (18). Visual funnel plots, Begg's test, and Egger's test were used to assess the risk of publication bias. Sensitivity analysis also assessed the stability of the results by ignoring each individual study. All statistical analyses were performed using Revman and Stata software.

## Results

### Literature search and included studies

**Figure 1:**
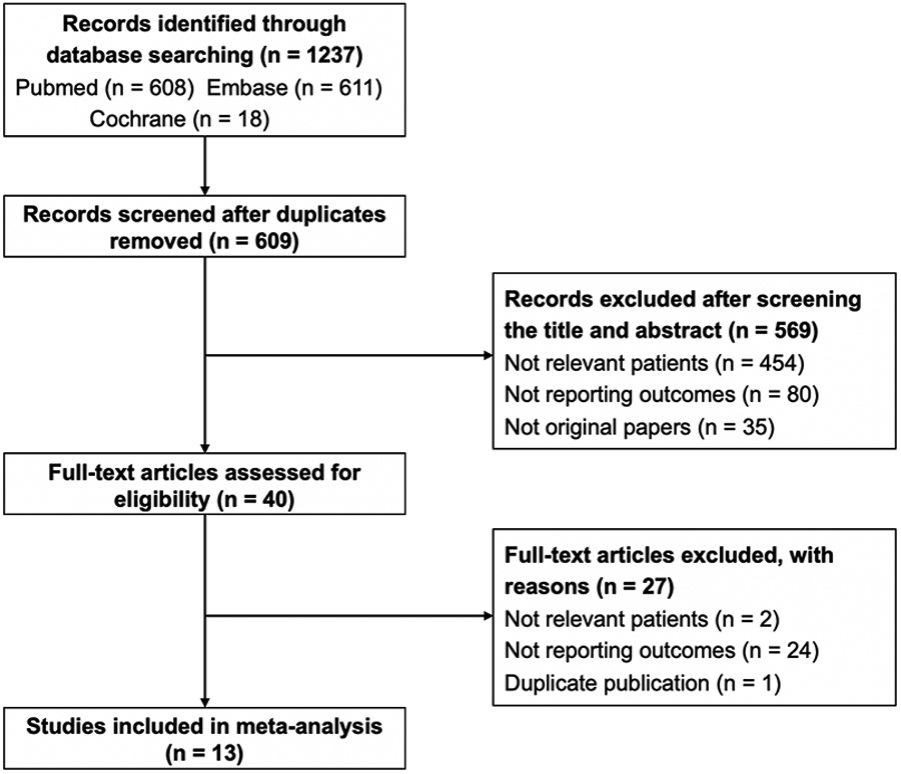
Flowchart of study selection process.

The detailed process of the literature search is presented in Figure 1. Through searching the three databases, 1,237 records were identified. After removing duplicates, 628 records were screened. After title and abstract were checked, 40 studies were carefully assessed with full text. Twenty-seven articles were excluded due to irrelevant patients, absence of outcomes, and duplication. Finally, 13 studies were included for analyses (2–4, 7, 9–15, 19, 20).

Due to the rarity of sRCC, the sample size of all included studies was small, ranged from 37 to 1,921. Seven studies were performed in the United States, five in China, and one in Korea. Two studies were retrospectively designed with multi-institutional data, two were prospectively designed with single-center data, and the others were retrospectively designed with single-center data. One study focused on nonmetastatic RCC, three studies focused on metastatic RCC, and the others focused on all stage RCC (Table 1). The score for study quality ranged from 6 to 8 (Supplementary Table S1).

**Table 1:** Overall characteristics of the included studies.

Studies	Study period	Country	Study design	Sample size	Age	Stage	Outcomes	Median follow-up (months)	SQ
Yang et al. (2021)	2004–2015	China	RTP, MI	1921	—	All	OS, CSS	13	7
Chahoud et al. (2021)	2013–2017	United States	RTP, SC	48	37 (31–72)^a^	M	OS	51.1	7
Zhao et al. (2020)	2007–2019	China	RTP, SC	139	60 (20–83)^a^	All	OS	20 (1–130)^a^	7
Silagy et al. (2020)	1989–2018	United States	PRO, SC	192	59 (52–66)	M	OS	14 (6.7–38.6)	8
Hou et al. (2020)	2010–2015	China	RTP, MI	428	63 (25–89)^a^	All	OS	8 (3–23)	7
Mano et al. (2019)	1994–2018	United States	PRO, SC	217	61 (52–69)	All	CSS, PFS	35.5/20.4	8
Wang et al. (2018)	2003–2017	China	RTP, SC	53	53 (43–58)	All	OS	NR	6
Thomas et al. (2016)	1986–2011	United States	RTP, SC	80	54 (49–64)	M	OS	NR	6
Gu et al. (2016)	2004–2015	China	RTP, SC	103	56 (16–79)^a^	All	OS	19.9 (10.8–35.1)	7
Zhang et al. (2015)	1970–2009	United States	RTP, SC	204	62 (32–88)^a^	All	CSS	NR	7
Merrill et al. (2015)	1986–2011	United States	RTP, SC	77	63 (38–85)^a^	NM	OS, PFS	20.4 (1–213.5)^a^	6
Park et al. (2013)	2001–2011	Korea	RTP, SC	37	58 (33–83)^a^	All	OS	23.1 (9.3–36.9)	6
Shuch et al. (2012)	1989–2009	United States	RTP, SC	104	59	All	OS	5.9 (4.7–8.9)	7

SQ, study quality according to the Newcastle-Ottawa scale; RTP, retrospective; SC, single center; PRO, prospective; MI, multi-institutional; All, nonmetastatic and metastatic; M, metastatic; NM, nonmetastatic; OS, overall survival; CSS, cancer-specific survival; PFS, progression-free survival; NR, not reported.

^a^ Median (range).

### Overall survival

**Figure 2:**
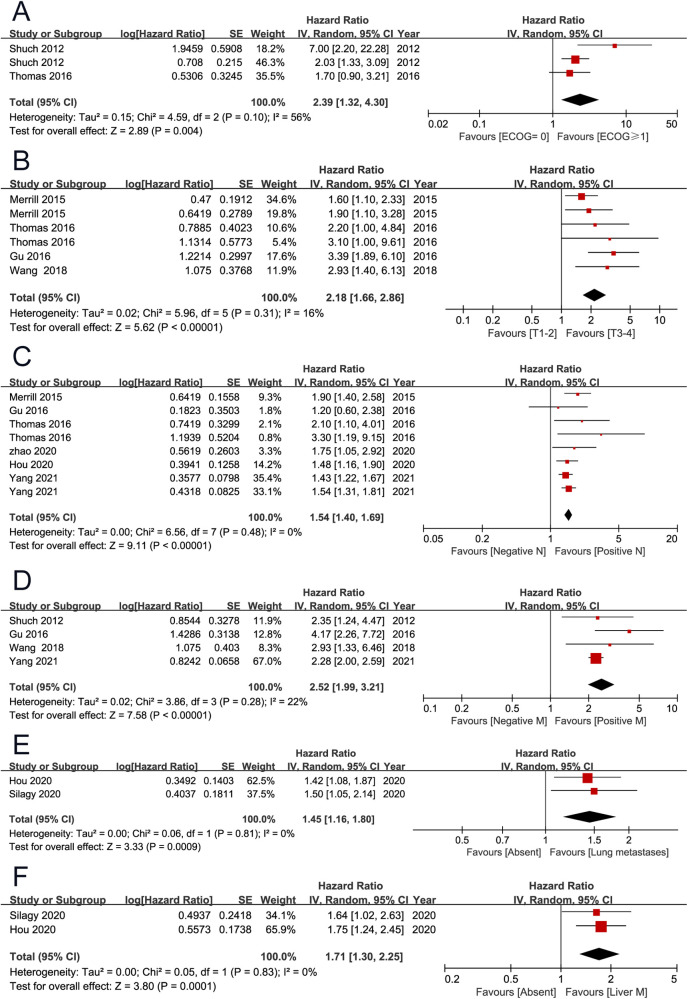
Forrest plots of meta-analyses of predictors of overall survival: (**A**) ECOG PS (≥1 vs 0); (**B**) T stage (3–4 vs 1–2); (**C**) positive lymph node; (**D**) distant metastasis; (**E**) lung metastases; (**F**) liver metastases. ECOG PS, European Cooperative Oncology Group performance score.

**Figure 3:**
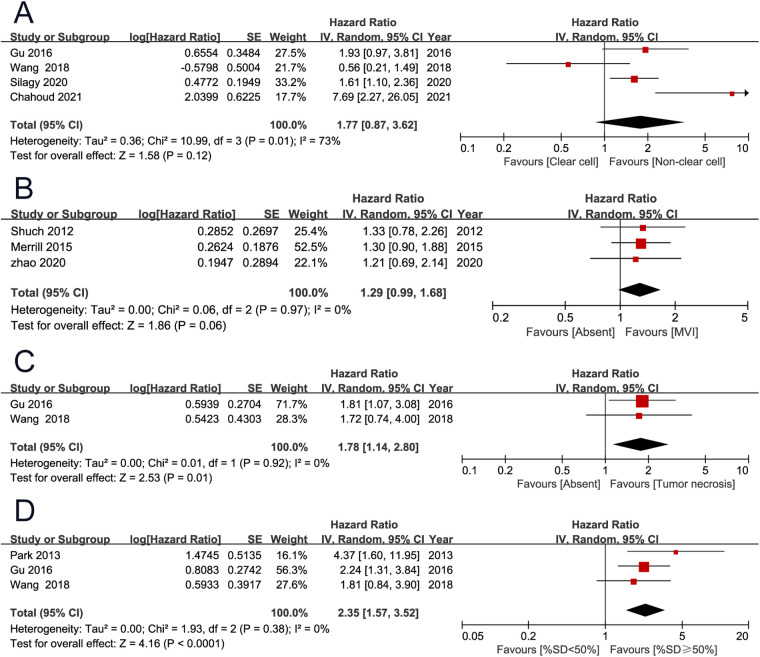
Forrest plots of meta-analyses of predictors of overall survival: (**A**) histology (non-clear cell vs clear cell); (**B**) microvascular invasion; (**C**) tumor necrosis; (**D**) percentage sarcomatoid (≥50% vs <50%).

OS was the primary endpoint, which was reported in 11 studies (2, 3, 7, 9–11, 13–15, 19, 20). The pooled results showed that high European Cooperative Oncology Group (ECOG) performance score (PS) (HR 2.39, 95% CI 1.32–4.30; *P* = 0.004), high T stage (HR 2.18, 95% CI 1.66–2.86; *P* < 0.001), positive lymph node (HR 1.54, 95% CI 1.40–1.69; *P* < 0.001), distant metastasis (HR 2.52, 95% CI 1.99–3.21; *P* < 0.001), lung metastases (HR 1.45, 95% CI 1.16–1.80; *P* < 0.001), liver metastases (HR 1.71, 95% CI 1.30–2.25; *P* < 0.001), tumor necrosis (HR 1.78, 95% CI 1.14–2.80; *P* = 0.010), and percentage sarcomatoid ≥50% (HR 2.35, 95% CI 1.57–3.52; *P* < 0.001) were associated with unfavorable overall survival. However, non-clear cell histology (HR 1.77, 95% CI 0.87–3.62; *P* = 0.120), and microvascular invasion (HR 1.29, 95% CI 0.99–1.68; *P* = 0.060) were not found to be associated with patient prognosis (Figures 2, 3) (Table 2).

**Table 2:** The pooled results of oncologic outcomes.

Variable	Studies	Pooled HR (95% CI)	*P* value	Heterogeneity *I*^2^ (%)	*P* value
Overall survival					
ECOG PS (≥1 vs 0)	3	2.3 9(1.32–4.30)	0.004	56	0.10
T stage (3–4 vs 1–2)	6	2.18 (1.66–2.86)	<0.001	16	0.31
Positive lymph node	8	1.54 (1.40–1.69)	<0.001	0	0.48
Distant metastasis	4	2.52 (1.99–3.21)	<0.001	22	0.28
Lung metastases	2	1.45 (1.16–1.80)	<0.001	0	0.81
Liver metastases	2	1.71 (1.30–2.25)	<0.001	0	0.83
Non-clear cell histology	4	1.77 (0.87–3.62)	0.120	73	0.01
Microvascular invasion	3	1.29 (0.99–1.68)	0.060	0	0.97
Tumor necrosis	2	1.78 (1.14–2.80)	0.010	0	0.92
Percentage sarcomatoid ≥50%	3	2.35 (1.57–3.52)	<0.001	0	0.38
Cancer-specific survival					
Positive lymph node	4	1.57 (1.33–1.85)	<0.001	47	0.13
NLR	2	1.16 (1.04–1.29)	0.008	55	0.14
Progression-free survival					
T stage (3–4 vs 1–2)	3	1.93 (1.44–2.58)	<0.001	0	0.70

HR, hazard ratio; CI, confidence interval; ECOG PS, European Cooperative Oncology Group performance score; NLR, neutrophil count to lymphocyte count.

### Cancer-specific survival and progression-free survival

**Figure 4:**
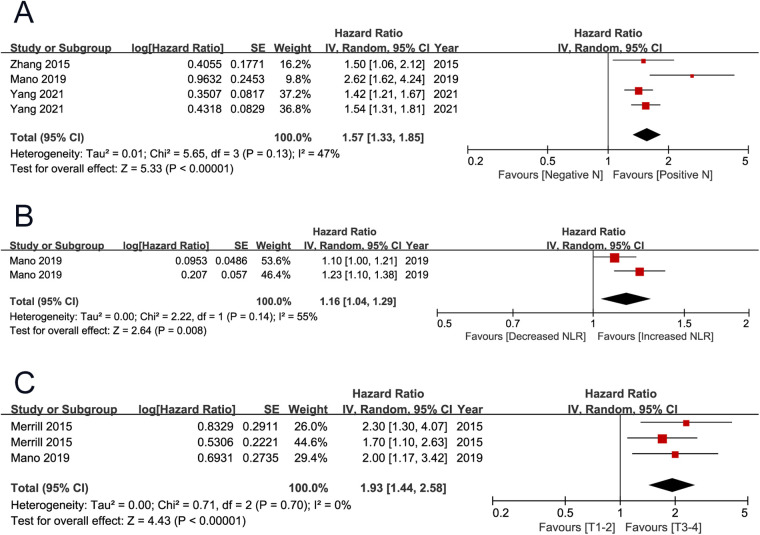
Forrest plots of meta-analyses of predictors of cancer-specific survival and progression-free survival: (**A**) positive lymph node; (**B**) NLR; (**C**) T stage (3–4 vs 1–2). NLR, neutrophil to lymphocyte ratio.

CSS and PFS were secondary endpoints, which were reported by three (4, 12, 19) and two (7, 12) studies. The pooled results showed that positive lymph node (HR 1.57, 95% CI 1.33–1.85; *P* < 0.001) and high neutrophil to lymphocyte ratio (NLR) (HR 1.16, 95% CI 1.04–1.29; *P* = 0.008) were associated with unfavorable cancer-specific survival. The pooled results showed that high T stage (HR 1.93 95% CI 1.44–2.58; *P* < 0.001) was associated with unfavorable progression-free survival (Figure 4) (Table 2).

### Publication bias and sensitivity analysis

**Figure 5:**
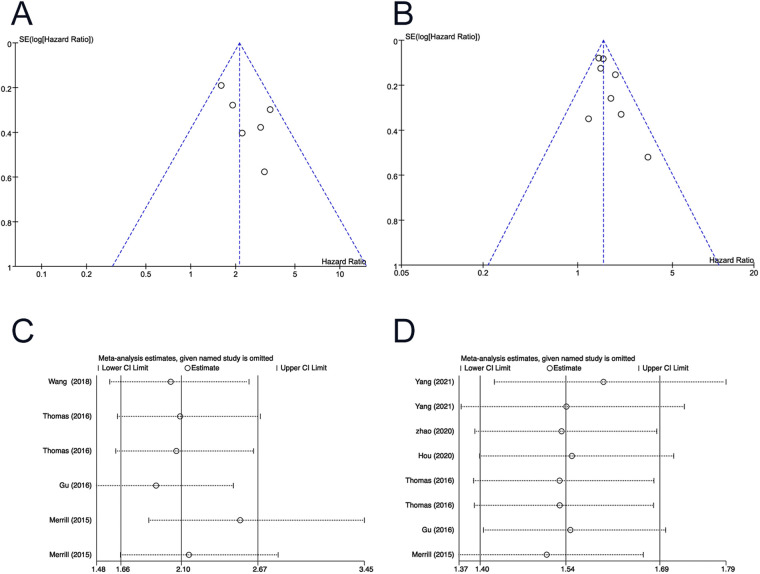
Funnel plot for (**A**) T stage (3–4 vs 1–2) of OS; (**B**) positive lymph node of OS. Sensitivity analysis for (**C**) T stage (3–4 vs 1–2) of OS; (**D**) positive lymph node of OS. OS, overall survival.

The funnel plots seemed to be symmetrical, and no statistical differences were identified (*P* > 0.05 for all). Sensitivity analyses confirmed the robustness of the results (Figure 5).

### Quality assessment

The evidence quality evaluation for each comparison with GRADE system is shown in Table 3. There were 13 comparisons. Certainty was very low in histology of OS and was low in microvascular invasion of OS. It was moderate for other comparisons.

**Table 3:** Grading of Recommendations Assessment, Development and Evaluation (GRADE) quality assessment of evidence for each comparison.

Certainty assessment							Effect			
Number of studies	Study design	Risk of bias	Inconsistency	Indirectness	Imprecision	Other considerations	Relative (95% CI)	Absolute (95% CI)	Certainty	Importance
OS—ECOG PS (≥1 vs 0)										
3	Observational studies	Not serious	Not serious	Not serious	Not serious	Strong association	HR 2.39 (1.32–4.30)	2 fewer per 1,000 (from 4 fewer to 1 fewer)	⊕⊕⊕◯ MODERATE	CRITICAL
OS—T stage (3–4 vs 1–2)										
6	Observational studies	Not serious	Not serious	Not serious	Not serious	Strong association	HR 2.18 (1.66–2.86)	2 fewer per 1,000 (from 3 fewer to 2 fewer)	⊕⊕⊕◯ MODERATE	CRITICAL
OS—positive lymph node										
8	Observational studies	Not serious	Not serious	Not serious	Not serious	Strong association	HR 1.54 (1.40–1.69)	2 fewer per 1,000 (from 2 fewer to 1 fewer)	⊕⊕⊕◯ MODERATE	CRITICAL
OS—distant metastasis										
3	Observational studies	Not serious	Not serious	Not serious	Not serious	Strong association	HR 2.52 (1.99–3.21)	2 fewer per 1,000 (from 3 fewer to 2 fewer)	⊕⊕⊕◯ MODERATE	CRITICAL
OS—lung metastases										
2	Observational studies	Not serious	Not serious	Not serious	Not serious	Strong association	HR 1.45 (1.16–1.80)	1 fewer per 1,000 (from 2 fewer to 1 fewer)	⊕⊕⊕◯ MODERATE	CRITICAL
OS—liver metastases										
2	Observational studies	Not serious	Not serious	Not serious	Not serious	Strong association	HR 1.71 (1.30–2.25)	2 fewer per 1,000 (from 2 fewer to 1 fewer)	⊕⊕⊕◯ MODERATE	CRITICAL
OS—histology (non-clear cell vs clear cell)										
4	Observational studies	Not serious	Serious	Not serious	Serious	None	HR 1.77 (0.87–3.62)	2 fewer per 1,000 (from 4 fewer to 1 fewer)	⊕◯◯◯ VERY LOW	CRITICAL
OS—microvascular invasion										
3	Observational Studies	Not serious	Not serious	Not serious	Serious	None	HR 1.29 (0.99–1.68)	1 fewer per 1,000 (from 2 fewer to 1 fewer)	⊕⊕◯◯ LOW	CRITICAL
OS—tumor necrosis										
2	Observational studies	Not serious	Not serious	Not serious	Not serious	Strong association	HR 1.78 (1.14–2.80)	2 fewer per 1,000 (from 3 fewer to 1 fewer)	⊕⊕⊕◯ MODERATE	CRITICAL
OS—percentage sarcomatoid (≥50% vs <50%)										
3	Observational studies	Not serious	Not serious	Not serious	Not serious	Strong association	HR 2.35 (1.57–3.52)	2 fewer per 1,000 (from 4 fewer to 2 fewer)	⊕⊕⊕◯ MODERATE	CRITICAL
CSS—positive lymph node										
4	Observational studies	Not serious	Not serious	Not serious	Not serious	Strong association	HR 1.57 (1.33–1.85)	2 fewer per 1,000 (from 2 fewer to 1 fewer)	⊕⊕⊕◯ MODERATE	CRITICAL
CSS—NLR										
2	Observational studies	Not serious	Not serious	Not serious	Not serious	Strong association	HR 1.16 (1.04–1.29)	1 fewer per 1,000 (from 1 fewer to 1 fewer)	⊕⊕⊕◯ MODERATE	CRITICAL
PFS—T stage (3–4 vs 1–2)										
3	Observational studies	Not serious	Not serious	Not serious	Not serious	Strong association	HR 1.93 (1.44–2.58)	2 fewer per 1,000 (from 3 fewer to 1 fewer)	⊕⊕⊕◯ MODERATE	IMPORTANT

CI, confidence interval; OS, overall survival; CSS, cancer-specific survival; NLR, neutrophil to lymphocyte ratio; PFS, progression-free survival; HR, hazard ratio; ECOG PS, European Cooperative Oncology Group performance score.

## Discussion

In clinical practice, sarcomatoid dedifferentiation has been shown to be an adverse predictor of survival in patients with RCC. sRCC is a highly aggressive form of cancer that represents 20% of advanced disease patients (5, 6). In addition, around 70% of patients with sRCC experience metastatic disease (3). A number of studies have reported median survival after diagnosis of sRCC patients ranging from 4 to 19 months (3, 8, 21, 22). However, there were significant differences in individual survival. A number of institutions have studied prognostic factors in patients with sRCC. Due to the limitations in study design and inconsistent results, we conducted this systematic review and meta-analysis.

Due to the rarity of sRCC, the sample size of all included studies was small, ranging from 37 to 1,921. Only two studies were conducted based on multi-institutional data, and the remaining studies were performed with single-center data. To control confounding factors and selection bias, we extracted only the results of the multivariate analyses reported in the literature. Most of the studied variables were clinical and pathological variables. Variable types and cut-off values are an important issue when merging data. Different studies may analyze the same variable in different types and cut-off values. For example, tumor size was studied as a continuous variable in one study (3), but it was studied as categorical variable with a cut-off of 7 cm in another study (14). Moreover, in these included studies, age and ECOG PS have been classified by different cut-off values, and T stage has been analyzed with a different classification method. The inconsistency precluded the data merging. In order to solve this, we used the most common classification method for these variables.

The endpoints included OS, CSS, and PFS. Merged results indicated that ECOG PS ≥1, stage T3–T4, positive lymph node, distant metastasis, lung metastases, liver metastases, tumor necrosis, and percentage of sarcomatoid dedifferentiation ≥50% were independent adverse prognostic factors for OS. Besides these significant variables, histology type was also an important prognosis predictor studied by several publications. In 192 sRCC patients with metastatic disease, Silagy et al. (10) found that the non-clear cell subtype experienced a significant poor survival compared with clear cell subtype, and non-clear cell histology (HR 1.61 95% CI 1.10–2.35; *P* = 0.010) was an independent adverse prognostic factor for OS. Also, in metastatic sRCC, Chahoud et al. found that non-clear cell histology was an independent adverse factor (20). However, the significance of this variable has not been identified by the other two studies (13, 14). After merging these results, non-clear cell histology (HR 1.77, 95% CI 0.87–3.62; *P* = 0.120) was not found to be associated with patient prognosis. The adverse prognostic factors for CSS included stage T3–T4 and high NLR. Stage T3–T4 was the only adverse prognostic factor for PFS. Because these variables are readily available clinicopathological factors, they can be used to guide patient counseling, risk stratification, and treatment selection.

In terms of the treatment of sRCC, surgical resection can be a reasonable choice. Though many patients with localized RCC and sarcomatoid dedifferentiation may experience postoperative disease recurrence (7), nephrectomy has been shown to be an independent prognostic factor, providing a survival benefit, even for cases with metastatic or stage IV disease (23). In addition, Haas et al. (24) reported that more than 75% of patients with sarcomatoid characteristics obtained response with combined doxorubicin and gemcitabine chemotherapy. Patients with a higher proportion of sarcomatoid components were more likely to obtain response with the therapy strategy. Due to the rarity of sRCC, the clinical evidence for sarcomatoid RCC was limited. In the age of targeted therapy, many treatments extrapolated from clear cell RCC were used in patients with sRCC. However, these cases responded poorly to anti-VEGF and anti-mTOR targeted therapy, and disease control at 6 months is rarely achieved in isolated patients (25). Compared with sunitinib alone, it was reported that sunitinib in combination with gemcitabine can obtain improved response rates and survival. Hence, the key role of chemotherapy can be neglected. More recently, immunotherapy-based combination therapies have shown superior outcomes in patients with sRCC, opening the possibility of a redefinition of first-line therapy (26). One study found that patients with sRCC treated with atezolizumab combined with bevacizumab had better PFS compared with patients receiving sunitinib (27). The Checkmate214 trial found that ipilimumab and nivolumab achieved a well-pleasing response rate to sRCC. In basic research, the genomic characteristics of sRCC have been analyzed in several studies. Higher rates of chromosome imbalance and more special somatic mutations in oncogenes were found in sarcomatoid elements (28, 29). These findings may provide insights into future treatment regimens.

The present study does have several limitations. First, due to the rarity of sRCC, the sample size of all included studies was small, ranging from 37 to 1,921. Totally, only 3,603 patients with sRCC were included in the current study. Two studies were performed based on multi-institutional databases, and the other 11 were conducted based on single-center data. The limited patients and selection basis from single-center data may affect the results, although only results from multivariate analyses were merged. Second, most of the studied variables were clinical and pathological variables. Some blood biomarkers, such as platelet to lymphocyte ratio and lymphocyte to monocyte ratio, have been reported by studies; however, the limited results preclude merging of the data. Third, though there were so many literature studies focusing on the prognostic factors of patients with sRCC, the studies that met our inclusion criteria were insufficient, especially for the endpoints of CSS and PFS. Only two and one variable have been analyzed for CSS and PFS, respectively. Fourth, potentially different criteria for assessment of sarcomatoid dedifferentiation (30), tumor necrosis, lymphovascular invasion, etc., may affect the results. Despite the above limitations, the present study initially evaluated the prognosis predictors for sRCC using the method of systematic review and meta-analysis. Based on the published literature, the present study can provide relatively comprehensive and reliable results about this issue.

In conclusion, based on a meta-analysis of available data, we identified the important prognostic factors of CSS, OS, and PFS in patients with sRCC. Therefore, these important factors should be systematically evaluated to suggest a risk-adaptive approach to patient counseling, risk stratification, and treatment selection.

## Data Availability

The original contributions presented in the study are included in the article/Supplementary Material, further inquiries can be directed to the corresponding author.
