# Supplementary material for: Clinicopathologic factors linked to oncologic outcomes for renal cell carcinoma with sarcomatoid dedifferentiation: A PRISMA-compliant systematic review and meta-analysis
Source: Front Surg. 2022 Oct 21;9:922150. doi: 10.3389/fsurg.2022.922150 (PMC9633959; doi:10.3389/fsurg.2022.922150)
Supplement: Supplementary file 1 [file Table1.docx]

**Table S1.** Newcastle-Ottawa Scale for risk of bias assessment of studies included in the meta-analysis.

| Studies | Selection | | | | Comparability | Outcome | | | Overall |
| --- | --- | --- | --- | --- | --- | --- | --- | --- | --- |
|  | Representativeness of exposed cohort | Selection of nonexposed | Ascertainment of exposure | Outcome not present at start |  | Assessment of outcome | Adequate follow-up length | Adequacy of follow-up |  |
| Yang (2021) | ★ | ★ | ★ | ★ | ★ | ★ | ☆ | ★ | 7 |
| Chahoud (2021) | ☆ | ★ | ★ | ★ | ★ | ★ | ★ | ★ | 7 |
| Zhao (2020) | ★ | ★ | ★ | ★ | ★ | ★ | ☆ | ★ | 7 |
| Silagy (2020) | ★ | ★ | ★ | ★ | ★ | ★ | ★ | ★ | 8 |
| Hou (2020) | ★ | ★ | ★ | ★ | ★ | ★ | ☆ | ★ | 7 |
| Mano (2019) | ★ | ★ | ★ | ★ | ★ | ★ | ★ | ☆ | 8 |
| Wang (2018) | ☆ | ★ | ★ | ★ | ★ | ★ | ★ | ☆ | 6 |
| Thomas (2016) | ☆ | ★ | ★ | ★ | ★ | ★ | ★ | ☆ | 6 |
| Gu (2016) | ★ | ★ | ★ | ★ | ★ | ★ | ☆ | ★ | 7 |
| Zhang (2015) | ★ | ★ | ★ | ★ | ★ | ★ | ★ | ☆ | 7 |
| Merrill (2015) | ☆ | ★ | ★ | ★ | ★ | ★ | ☆ | ★ | 6 |
| Park (2013) | ☆ | ★ | ★ | ★ | ★ | ★ | ☆ | ★ | 6 |
| Shuch (2012) | ★ | ★ | ★ | ★ | ★ | ★ | ☆ | ★ | 7 |
